# HPV knowledge and non-adherence to cervical cancer screening before and following the COVID-19 pandemic in the United States

**DOI:** 10.1371/journal.pgph.0004800

**Published:** 2025-09-10

**Authors:** Mariah Malak Bilalaga, Greeshma Gaddipati, Anirudra Devkota, Ramya Vasireddy, Ted Akhiwu, Joseph Atarere, Boniface Mensah, Christopher Haas, Louay Almidani

**Affiliations:** 1 MedStar Health Georgetown University Internal Medicine Residency Program, Baltimore, Maryland, United States of America; 2 Wilmer Eye Institute, Johns Hopkins University, Baltimore, Maryland, United States of America; Babcock University, NIGERIA

## Abstract

To compare HPV vaccination knowledge and non-adherence rates to cervical cancer screening in a nationally representative sample of American women before and following the COVID-19 pandemic, female participants aged 21–65 years from the National Cancer Institute Health Information National Trends Survey 2019 and 2022 were included. Adherence to cervical cancer screening was assessed based on the timing of their last Papanicolaou (PAP) smear, with participants classified as non-adherent to cervical cancer guidelines if their last PAP smear was > 3 years. Further, participants were asked about their knowledge of the HPV vaccine and were categorized as unaware if they had not heard of it before. Survey-weighted Poisson regression models adjusted for age, race, and education were used to explore both adherence and HPV vaccination knowledge before and following the COVID-19 pandemic. We included 1,905 females with a mean age of 44.5 years, 61.3% were White, and 35.4% had a college education or higher. The prevalence of non-adherence in 2019 was 19.2% and increased by 6.6% in 2022. White adults showed an increase of 5.6%, while Black adults showed an increase of 13.9%. When exploring changes in HPV vaccination knowledge, 18.9% did not hear about the HPV vaccine in 2019, which slightly increased by 2.8% in 2022. Being Black and having lower education were significantly associated with both greater non-adherence rates and not hearing about HPV vaccine. Non-adherence to cervical cancer screening increased following the COVID-19 pandemic, especially amongst under-represented communities including Black adults and those with lower education. This trend was also reflected in reduced awareness of HPV vaccination. Further studies are needed to elucidate barriers associated with greater non-adherence rates and to explore targeted interventions, such as educational campaigns, community outreach programs, and initiatives to improve access for underserved populations, which may promote more equitable screening uptake and healthcare access.

## Introduction

Cervical cancer is the fourth most common cancer among females worldwide, following lung, colorectal, and breast cancer [1]. Although the incidence and mortality of cervical cancer have been increasing in low-income countries, the incidence has been decreasing in developed countries due to the widespread implementation of cytological screening programs [1,2]. This is likely because screening allows for the early detection and treatment of precancerous lesions, thereby preventing progression to invasive cervical cancer and ultimately reducing disease incidence.

Screening for cervical cancer is a cost-effective approach to reducing both the global and individual burden of the disease [3]. The United States Preventative Service Task Force (USPTF) recommends cervical cancer screening with cytology every three years for females aged 21–29, while for women aged 30–65, it is recommended to either: 1) screen with cytology every three years 2) with a combination of cytology and human papillomavirus testing every five years, or 3) with HPV testing alone every five years [4]. The recommendations for women over the age of 65 years state that if the woman has an adequate screening history with negative results, then no further screening is warranted [4].

Nonetheless, various aspects of healthcare delivery were disrupted by the COVID-19 pandemic and have not yet recovered to their pre-pandemic levels of operation, including preventative healthcare [5]. Limited studies explored how screening rates changed in a population-based sample following the pandemic, with varying findings [6–8].

Further, the HPV vaccine, introduced in 2006 in the United States, created important opportunities to reduce HPV-related disease burden, Given general vaccine hesitancy following the pandemic, understanding whether knowledge about HPV vaccination has changed post-pandemic is important to guide public health planning.

Disruptions caused by the pandemic have disproportionately impacted minorities who already face systemic barriers to healthcare access, including limited access to preventative services [9]. According to Andersen’s Behavioral Model, socioeconomic status and access to care can shape health behaviors by influencing individuals’ ability to obtain and engage in recommended preventive behaviors, such as cervical cancer screening [10]. As such, understanding these disparities as well as other factors associated with lower adherence rates is critical for developing targeted interventions aimed at improving these services among underrepresented populations.

Here, using a nationally representative sample of non-institutionalized (community-dwelling individuals living outside of nursing homes or other institutional settings) American women, the objective of this study is to quantify both the non-adherence rates to cervical cancer screening and knowledge about HPV vaccinations before and following the onset of the COVID-19 pandemic. Specifically, we sought to answer the following research questions: How have cervical cancer screening rates and HPV vaccination knowledge changed following the pandemic? What factors are associated with these changes? By addressing these questions, our study aims to elucidate trends in HPV vaccination knowledge and cervical cancer screening adherence in the U.S. before and after the COVID-19 pandemic.

## Methods

### Study design and participants

This population-based repeated cross-sectional study used data from the National Cancer Institute Health Information National Trends Survey (HINTS) rounds 5 (2019) and 6 (2022) to explore the impact of COVID-19 on cervical cancer non-adherence rates and knowledge about HPV vaccine [11]. HINTS is a postal survey that is conducted every two years, reaching a diverse sample of non-institutionalized American adults [11]. Participants were selected through a multi-stage stratified random sampling approach from a nationwide household address list. Households receiving the surveys are randomly selected using address-based sampling. The sample was restricted to female adults aged 21–65 years with no self-reported history of cervical cancer. This age range was chosen in accordance with the USPSTF Grade A recommendations for cervical cancer screening [4]. Data were analyzed from March 2024 to August 2024.

### Ethics statement

For this study, approval from an institutional review board was not required as it uses publicly available, nonidentifiable data. The HINTS investigators obtained consent from all participants (through return of the survey). This study followed the Strengthening the Reporting of Observational Studies in Epidemiology (STROBE) reporting guideline (S1 Checklist).

### Outcomes

Adherence to cervical cancer screening was assessed based on the timing of their last PAP smear test (self-reported). Participants were classified as non-adherent to cervical cancer guidelines if their last reported PAP smear test was more than three years ago. Further, we were interested in studying whether knowledge about HPV vaccination varied after the pandemic. Although HPV vaccine is not recommended for women after the age of 26 years, we included older age groups (till the age of 65 years) in our analysis because we were interested in understanding public knowledge about the vaccine, which is not only relevant for vaccine uptake but also reflects broader health awareness. This may influence intergenerational communication and support for public health initiatives. Specifically, older individuals (such as parents, caregivers, or healthcare decision-makers) may play a significant role in influencing the vaccination decisions of eligible younger populations. As such, we opted to include all age groups to capture a more comprehensive picture of public knowledge and assess whether the pandemic had a differential impact on awareness. Participants were asked about their knowledge of the HPV vaccine and were categorized as unaware if they indicated they had not heard of it before.

### Covariates

Age, race and ethnicity, education, and frequency of going to a healthcare provider in the past twelve months were recorded. Race and ethnicity were categorized as non-Hispanic White, non-Hispanic Black, Hispanic, and Other [which included American Indian or Alaskan Native, Native Hawaiian or Pacific Islander, Asians, and multiple races]. Education was categorized as <high school, completed high school, some college, and ≥ college graduate. Frequency of going to a healthcare provider was defined based on answers to the following question: “In the past 12 months, not counting times you went to an emergency room, how many times did you go to a doctor, nurse, or other health professional to get care for yourself?”. Participants were categorized as none, one time, or two or more times. All covariates were based on self-report.

### Statistical analysis

Descriptive statistics were used to describe variables by year. Means and standard deviations (SD) were used to summarize continuous variables, while categorical variables were presented as counts and proportions. Multivariable Poisson regression models were used to explore the relationship between age, race and ethnicity, education, and provider frequency on cervical cancer non-adherence before and following the COVID-19 pandemic. Similar models were used with knowledge about HPV vaccine as the dependent variables. In sensitivity analysis, we repeated the models examining adherence and knowledge about the HPV vaccine, stratified by age (≤26 vs. > 26), as results may differ between women who were offered the vaccine and those who were not [12]. All analyses accounted for the complex survey design, including sampling weights, clustering of observations, and stratification, and used design-based variance estimation (equivalent to robust standard errors) to produce generalizable population-level estimates. “Don’t know” and “Refuse” responses were treated as missing values and excluded from the regressions. All P values were 2-sided (as the uncertainty of the effect’s direction and the study’s descriptive nature warranted the use of a two-tailed test) but not adjusted for multiple analyses. All analyses were performed using Stata version 16.1 (Stata Corp).

## Results

In 2019, among 1,905 female adults, representing a population of 92,019,982 female adults, the mean age (standard deviation [SD]) was 44.5 (12.6) years, 61.3% were non-Hispanic White, and 35.4% had a college education or higher. In 2022, among 2,194 female adults, representing a population of 87,170,765 female adults, the mean age (SD) was 44.4 (12.5) years, 56.5% were non-Hispanic White, and 37.9% had a college education or higher (Table 1).

**Table 1:** Demographics included participants in both rounds. Abbreviations; SD: standard deviation. n is unweighted, % is survey-weighted estimates.

	Round 5 (2019)	Round 6 (2022)
**Participants, n**	1,905	2,194
**Age in years, weighted mean (SD)**	44.5 (12.6)	44.4 (12.5)
**Race and ethnicity, n (%)**		
Non-Hispanic White	1,028 (61.3)	1,086 (56.5)
Non-Hispanic Black	312 (12.5)	387 (13.1)
Hispanic	296 (18.4)	472 (19.3)
Other*	163 (7.9)	190 (11.2)
**Education, n (%)**		
< High school	106 (6.4)	127 (5.0)
Completed high school	274 (19.9)	362 (17.4)
Some college	526 (38.3)	607 (39.8)
≥ College graduate	996 (35.4)	1,092 (37.9)

*Other included American Indian or Alaskan Native, Native Hawaiian or Pacific Islander, Asians, and multiple races

Throughout the results, percentages represent raw values, while ratios represent adjusted coefficients from the regression models. In 2019, the prevalence of non-adherence to cervical cancer screening was 19.2% [95% CI: 16.3, 22.5]. In adjusted models (Table 2), being non-Hispanic Black (prevalence ratio [PR] = 0.47 [95% CI: 0.29, 0.75] vs. non-Hispanic White) had lower non-adherence rates. Greater frequency of visiting a healthcare provider (PR = 0.35 [95% CI: 0.24, 0.53]; 2 + visits vs. none) was associated with lower likelihood of non-adherence. In 2022, the prevalence of non-adherence to cervical cancer screening was 25.8% [95% CI: 22.9, 29.1]. In adjusted models, having lower education was associated with 1.63 [95% CI: 1.00, 2.67] higher likelihood of non-adherence to cervical cancer screening guidelines, while age and race were not (p > 0.05). Greater frequency of visiting a healthcare provider (PR = 0.58 [95% CI: 0.42, 0.81]; 2 + visits vs. none) remained associated with lower likelihood of non-adherence. Compared to 2019, non-adherence rates increased by 6.6% in 2022. When stratified by race, non-Hispanic White adults showed an increase of 5.6% [95% CI: 5.4, 5.6] in non-adherence rates, while non-Hispanic Black adults showed an increase of 13.9% [95% CI: 12.0, 15.2].

**Table 2:** Multivariable models exploring predictors of non-adherence to cervical cancer screening.

Predictor	Interval/Reference	Cervical cancer non-adherence					
		Round 5 (2019)			Round 6 (2022)		
		PR	95% CI	p-value	PR	95% CI	p-value
**Age**	1 year older	0.99	(0.98, 1.01)	0.43	1.00	(0.99, 1.01)	0.82
**Race**							
Non-Hispanic Black	Non-Hispanic White	0.47	(0.29, 0.75)	0.002	0.93	(0.68, 1.28)	0.65
Hispanic	Non-Hispanic White	0.80	(0.45, 1.44)	0.45	0.87	(0.63, 1.21)	0.40
Other*	Non-Hispanic White	1.15	(0.76, 1.74)	0.51	1.11	(0.67, 1.84)	0.68
**Education**							
< High school	≥ College graduate	1.71	(0.95, 3.08)	0.07	1.63	(1.00, 2.67)	0.05
Completed high school	≥ College graduate	1.68	(1.14, 2.47)	0.01	1.23	(0.89, 1.71)	0.21
Some college	≥ College graduate	1.58	(1.09, 2.29)	0.02	1.06	(0.82, 1.38)	0.65
**Frequency of healthcare visits**							
1	0	0.49	(0.27, 0.88)	0.02	0.88	(0.57, 1.35)	0.54
2+	0	0.35	(0.24, 0.53)	<0.001	0.58	(0.42, 0.81)	0.002

Abbreviations: PR: prevalence ratio; CI: confidence interval.

*Other included American Indian or Alaskan Native, Native Hawaiian or Pacific Islander, Asians, and multiple races

When exploring changes in HPV vaccination knowledge,18.9% [95% CI: 16.0, 22.1] did not hear about the HPV vaccine in 2019, while in 2022, 21.7% [95% CI: 18.5%, 25.3%] were unaware about HPV vaccine. HPV vaccine awareness declined slightly (by 2.8%) in 2022 compared to 2019, though this change was not statistically significant. When stratified by race, non-Hispanic White adults showed an increase in knowledge about HPV vaccine by 2.5%, while Black adults showed a decrease by 9.4%. When exploring predictors of HPV knowledge, older age (PR = 1.02 [95% CI: 1.005, 1.03]; per 1 year older), being Black (PR = 1.49 [95% CI: 1.03, 2.17]; vs. White), and having lower education (PR = 3.31 [95% CI: 2.08, 5.27]; less than high school vs. college graduate or higher) were significantly associated with not hearing about HPV vaccine in 2019. Being Black (PR = 2.51 [95% CI: 1.62, 3.89]; vs. White) and having lower education (PR = 2.55 [95% CI: 1.70, 3.83]; less than high school vs. college graduate or higher) remained significantly associated with not hearing about HPV vaccine in 2022 (Table 3).

**Table 3:** Multivariable models exploring predictors of not hearing about HPV vaccine.

Predictor	Interval/Reference	Did not hear about HPV vaccine					
		Round 5 (2019)			Round 6 (2022)		
		PR	95% CI	p-value	PR	95% CI	p-value
**Age**	1 year older	1.02	(1.005, 1.03)	0.01	1.01	(1.00, 1.02)	0.09
**Race**							
Non-Hispanic Black	Non-Hispanic White	1.49	(1.03, 2.17)	0.04	2.51	(1.62, 3.89)	<0.001
Hispanic	Non-Hispanic White	1.54	(1.00, 2.38)	0.05	2.73	(1.81, 4.1)	<0.001
Other	Non-Hispanic White	2.34	(1.35, 4.07)	0.003	3.68	(2.12, 6.38)	<0.001
**Education**							
< High school	≥ College graduate	3.31	(2.08, 5.27)	<0.001	2.55	(1.70, 3.83)	<0.001
Completed high school	≥ College graduate	2.79	(1.87, 4.15)	<0.001	2.44	(1.66, 3.60)	<0.001
Some college	≥ College graduate	1.25	(0.87, 1.82)	0.23	1.60	(1.08, 2.38)	0.02
**Frequency of healthcare visits**							
1	0	0.78	(0.44, 1.38)	0.38	0.70	(0.44, 1.10)	0.12
+	0	0.86	(0.55, 1.33)	0.49	0.68	(0.47, 0.99)	0.05

Abbreviations: PR: prevalence ratio; CI: confidence interval.

*Other included American Indian or Alaskan Native, Native Hawaiian or Pacific Islander, Asians, and multiple races

In sensitivity analyses exploring adherence differences by age group, 27.7% [95% CI: 15.7, 43.9] of women aged ≤26 years were not adherent to cervical cancer screening in 2019, compared to 43.0% [95% CI: 27.1%, 60.6%] in 2022; i.e., HPV nonadherence increased by 15.3% in adults aged ≤26 years, though this change was not statistically significant. When looking at adults >26 years, 18.0% [95% CI: 16.9, 23.1] of women aged >26 years were not adherent to cervical cancer screening in 2019, compared to 23.7% [95% CI: 21.1%, 26.4%] in 2022. Cervical cancer screening nonadherence increased by 5.7% in 2022 compared to 2019 in adults aged >26 years, though this change was not statistically significant either.

In sensitivity analyses exploring vaccination awareness differences by age group, 11.6% [95% CI: 6.8, 19.3] of women aged ≤26 years did not hear about the HPV vaccine in 2019, compared to 23.6% [95% CI: 13.0%, 38.9%] in 2022; i.e., HPV vaccine awareness declined by 12% in adults aged ≤26 years, though this change was not statistically significant. When looking at adults >26 years, 19.8% [95% CI: 16.9, 23.1] of women aged >26 years did not hear about the HPV vaccine in 2019, compared to 21.5% [95% CI: 18.2%, 25.2%] in 2022. HPV vaccine awareness declined slightly (by 1.7%) in 2022 compared to 2019 in adults aged >26 years, though this change was not statistically significant.

## Discussion

In a nationally representative sample of female adults, non-adherence to cervical cancer screening increased following the COVID-19 pandemic, particularly amongst higher risk groups including Black adults, those with lower education, and those with less frequent visits to a health provider. These findings suggest that racial/ethnic and socioeconomic disparities in cervical cancer screening and HPV vaccine awareness persisted post-pandemic, with certain groups facing greater healthcare access challenges, highlighting the need to identify methods to provide more equitable access to healthcare services.

Similar to our findings, Sabatino et al (2023) utilized data from the 2019 and 2021 National Health Interview Survey and showed that around 75% of US women were adherent to cervical cancer screening, with no differences in estimates before and following the pandemic [6]. Although they had a similar inclusion criteria to our work and found similar estimates, the differences in pre-pandemic estimates may be due to different populations assessed or differences in survey methods. In another study, Borders et al (2024) explored changes in adherence to cervical cancer and, similar to our findings, found lower PAP testing in 2022 compared to 2019, though they failed to exclude participants with cervical cancer, which may inaccurately reflect population-based PAP smear testing and did not explore whether changes over time varied by race or education [8]. Irrespective, harmonization of survey methods across national datasets is important to derive accurate estimates. Adherence rates are still below the target goals for cervical cancer screening and further work is needed to elucidate approaches to improve adherence.

When exploring predictors of adherence, we found that Black women had better levels of adherence pre-pandemic. Our results agree with previous work by Haas et al. (2012) who showed that women of Black and Hispanic decants were more likely to get screened for cervical cancer than Whites [13]. However, following the pandemic, the rates of non-adherence to cervical cancer screening were disproportionately higher for Blacks in our study. This is an especially important result suggesting that the pandemic increased barriers faced by minorities and marginalized female adults, which has been shown for healthcare services other than cervical cancer screening [14]. Further, when stratified by age groups, those aged 26 or younger displayed a greater increase in nonadherence rates compared to their counterparts following the pandemic, though did not reach statistical significance likely due to limited power. This finding, in participants most qualifying for the testing protocol, is concerning, and further highlights barriers faced by younger women. Approaches to optimize the health and inclusion of minorities and higher risk communities are important particularly post-pandemic.

Further, better education and greater frequency of healthcare visits were both significantly associated with improved adherence. However, the magnitude of these effects appeared smaller in 2022 compared to 2019, suggesting that the relationship between education, healthcare visits, and adherence may have been influenced by the pandemic. While this study could not directly assess effect modification, future work is needed to determine whether and how the pandemic altered these associations, which could inform targeted interventions to improve adherence following the pandemic.

We further found that knowledge about HPV vaccine dropped slightly, though not at a statistically significant level. However, when stratified by age groups, those aged 26 or younger displayed a greater drop in knowledge compared to their counterparts. This may be due to disruptions in health education and preventive care services during the pandemic, which disproportionately impacted younger individuals who may have otherwise received vaccine information through school-based or primary care outreach. On the other hand, older women may have had consistently lower levels of HPV vaccine knowledge overall, in part due to the timing of the vaccine’s introduction in 2006, which occurred after they were beyond the age of routine vaccination. This helps explain why older age was significantly associated with not having heard about the HPV vaccine in our models and underscores the importance of tailoring educational efforts across age groups to address gaps in vaccine awareness. Future age-period-cohort models using longitudinal datasets may be utilized to confirm this finding and disentangle this relationship.

Overall, our findings suggests that HPV knowledge was not affected by general vaccine acceptability post-pandemic. Yet, we found that around 20% (both pre- and post-pandemic) of the population have not heard about HPV vaccine with a higher likelihood of unawareness among Black adults and those with lower education. Previous literature reported higher estimates of around 35% [15], with notably lower awareness among individuals with less than a high school education and minorities [16]. As suggested by our findings, promoting more frequent and regular primary care provider visits and discussions of cancer screening is important to improve HPV knowledge and cervical cancer adherence, especially among higher risk groups.

Previous work suggested other approaches to improve adherence. Greater continuity of care, those who saw the same provider versus different providers, showed better adherence rates [13]. Similarly, physician-patient gender concordance, or those who have a female as their primary care provider, were more likely to be up to date on their cervical cancer screening [13]. Future work is needed to elucidate whether race and ethnicity concordance between patients and physicians may also improve their adherence.

Several barriers may lead to decreased or delayed adherence to cervical cancer screening [17]. Personal barriers include the fear of discovering cancer, shame, screening by a male doctor, ignorance of risk factors, recent immigration status, and the existence of chronic conditions [17–19]. Structural constraints involve needing time off work, lack of transportation and childcare, inadequate English proficiency, inconsistent doctor visits, and absence of a doctor’s recommendation [20]. Social and systemic challenges to cervical cancer screening disproportionately affect women from underserved communities in the United States, increasing the likelihood of underscreening. Medical mistrust, stemming from historical discrimination and negative healthcare experiences, can deter underserved communities from adhering to cervical cancer screening and seeking HPV vaccination. Overall, it is imperative to reduce barriers and improve healthcare services, including cancer screening, in these communities.

Our findings should be interpreted in the context of a few limitations. First, adherence and knowledge data were self-reported by the participants. It is possible that some participants may under- or over-estimate the timing of their last PAP test [21]. Second, data on HPV screening is unavailable in the HINTS dataset; some patients may have undergone the combined regimen of HPV screening along with PAP test every 5 years rather than PAP screening every 3 years, though a recent study using the MarketScan database found that PAP tests are the most common screening modality [22]. By studying a single approach for cervical cancer screening (cytology every three years), our findings may underestimate the overall prevalence of adherence to screening guidelines, though the direction and magnitude of change following the pandemic may be less affected. Third, we lack intermediate data for the years 2020–2021. Without data from these years, it is difficult to assess whether changes in screening rates and HPV vaccination knowledge occurred gradually or abruptly in response to the pandemic. Fourth, excluding institutionalized individuals may limit the generalizability of the findings, as this population may have different healthcare access and screening behaviors compared to community-dwelling adults. Fifth, knowledge is a complex construct, and hearing about the vaccine does not imply understanding. Sixth, around 15% of our participants were aged 26 years or younger, which are most qualifying for initiation of screening/vaccination; we may be underpowered to estimate significant changes following the pandemic. Seventh, we acknowledge the possibly of residual confounding. Future studies should incorporate more detailed questionnaires to better explore this relationship. Last, we acknowledge that our findings could be due to residual confounding such as insurance status which could not be accurately captured using self-reported data [13].

## Conclusion

In a nationally representative sample of female Americans, non-adherence to cervical cancer screening increased following the COVID-19 pandemic, especially amongst under-represented communities including Black adults and those with lower education. Further studies are needed to elucidate barriers associated with greater non-adherence rates and to explore targeted interventions, such as educational campaigns, community outreach programs, and initiatives to improve healthcare access for underserved populations, which may promote more equitable screening uptake and healthcare access.

## Supporting information

S1 Checklist STROBE Statement. (DOCX)
